# Prophylaxis of migraine with aura: a place for acetylsalicylic acid

**DOI:** 10.1186/1129-2377-14-S1-P195

**Published:** 2013-02-21

**Authors:** LT Savi, V Condello, F Bert, L Pinessi

**Affiliations:** 1 Headache Centre, Italy; 2 Department of Public Health, Italy; 3 University of Turin, Italy

## Aim

Aim of our study was to assess the efficacy and tolerability of Acetylsalicylic acid (ASA) in migraine with aura (MA) management, in a sample afferent to the Headache Centre of San Giovanni Battista University-Hospital of Torino.

## Materials and methods

We analyzed the medical records of 196 patients affering consecutively to our Centre between 1995 and 2007 and receiving a prophylactic treatment, dividing them in two groups: the ones receiving ASA (90) and those who were treated with other therapies (106). Primary endpoint was to evaluate the improvement in MA crisis frequency in the two groups. A binary logistic regression model was used to identify possible factors associated with the positive response to treatment.

## Results

The mean age was 32.1 (±9.9) in ASA group and 36.8 (±14.9) in no-ASA group. Positive response to treatment (measured as a reduction of at least the 50% of crisis with aura) was reported by 85.6% of patients in the ASA group and 51.9% in the control group (p< 0.001). Multivariate analysis showed, as only variable related with a positive response to treatment, the group (ASA Group: OR 6.26, p= 0.006), while there were no relationships with gender, age or typology of aura.

## Discussion

In the past, other studies compared the effectiveness of ASA in migraine versus other prophylactic therapies, but they often considered very small samples, mixing MA and migraine without aura (MoA) together. In those setting ASA appeared to be mildly effective. Our results show a large positive response to the treatment with Acetylsalicylic acid, whose probability of success was about six times greater than the one associated with other therapies.

## Conclusions

According to our results, asa is not only effective in the majority of MA cases, but the response is usually evident in a short time. A double blind study with a larger sample is needed to ascertain these findings.

